# Dementia risk in the general population: large-scale external validation of prediction models in the AGES-Reykjavik study

**DOI:** 10.1007/s10654-021-00785-x

**Published:** 2021-07-25

**Authors:** Jet M. J. Vonk, Jacoba P. Greving, Vilmundur Gudnason, Lenore J. Launer, Mirjam I. Geerlings

**Affiliations:** 1 Department of Epidemiology, Julius Center for Health Sciences and Primary Care, University Medical Center Utrecht, Stratenum 6.131, PO BOX 85500, 3508 GA Utrecht, The Netherlands; 2 Department of Neurology, College of Physicians and Surgeons, Taub Institute for Research On Alzheimer’s Disease and the Aging Brain, Columbia University, New York, NY USA; 3 Icelandic Heart Association, Kopavogur, Iceland; 4 University of Iceland, Reykjavík, Iceland; 5 Laboratory of Epidemiology and Population Sciences, Demography and Biometry, National Institute On Aging, National Institutes of Health, Gateway Building, Suite 3C-309, 7201 Wisconsin Ave., Bethesda, MD 20892 USA

**Keywords:** Dementia, Alzheimer’s disease, Prognosis, Validation

## Abstract

We aimed to evaluate the external performance of prediction models for all-cause dementia or AD in the general population, which can aid selection of high-risk individuals for clinical trials and prevention. We identified 17 out of 36 eligible published prognostic models for external validation in the population-based AGES-Reykjavik Study. Predictive performance was assessed with *c* statistics and calibration plots. All five models with a *c* statistic > .75 (.76–.81) contained cognitive testing as a predictor, while all models with lower *c* statistics (.67–.75) did not. Calibration ranged from good to poor across all models, including systematic risk overestimation or overestimation for particularly the highest risk group. Models that overestimate risk may be acceptable for exclusion purposes, but lack the ability to accurately identify individuals at higher dementia risk. Both updating existing models or developing new models aimed at identifying high-risk individuals, as well as more external validation studies of dementia prediction models are warranted.

**Supplementary Information:**

The online version contains supplementary material available at 10.1007/s10654-021-00785-x.

## Background

The global increase in prevalence and incidence of dementia—mainly due to prolonged life expectancy—carries substantial individual, societal, and economic burden [1]. With currently no available cure, development of potential intervention is reliant on accurate identification of asymptomatic individuals at high risk of being in the preclinical phase of the disease [2]. Multiple risk factors for all-cause dementia and Alzheimer’s disease (AD) have been identified, ranging from non-modifiable factors such as genetics (e.g., APOE e4) to modifiable medical (e.g., cardiovascular health) and environmental influences (e.g., level of education) [3–5]. These risk factors can be used to estimate someone’s individual probability of developing all-cause dementia or AD over a specified time through multivariable prognostic modeling. Accurate prognostic models can inform individuals and health professionals about individualized dementia risk, support personalized care, and aid selection of high-risk individuals for clinical trials and prevention [6–8].

Development of new models for dementia prediction has flourished across the past decades [9–12]. However, the majority of models has not been validated in a dataset other than the one it was developed in [10]. Model development and internal validation in one dataset typically lead to opportunistic predictive performance [13]. External validation of a model’s ability to differentiate who does and does not develop dementia (i.e., discrimination) and the model’s agreement between predicted and observed risks (i.e., calibration) in an independent dataset can quantify optimism due to overfitting or statistical modeling limitations [13]. Moreover, evaluating predictive models in another setting (e.g., health care, geographical, or cultural) than the one it was developed in, also called ‘transportability,’ can assess broader applicability of the model [14].

As external validation is essential to evaluate the generalizability and transportability of a prediction model, its lack thereof forms a major limitation for using prognostic scores in clinical practice and research [6]. This study aimed to evaluate the external performance of prediction models for all-cause dementia or AD in older adults that were developed for prediction horizons of 5–10 years. We validated these models in an independent population-based cohort: The Age, Gene/Environment Susceptibility—Reykjavik Study (AGES-RS).

## Methods

### Literature search, selection criteria, and screening process

Previous work on prediction models of dementia has been summarized in four extensive systematic reviews that cover prediction models generated up until April 1, 2018 [9–12]. Additionally, we searched PubMed for models published between January 1, 2018 and April 1, 2020 using the following search string: (model OR models) AND (risk) AND (dementia OR Alzheimer) AND (predict* AND (develop* OR create*)) AND (ROC OR (c statistic) OR (c-statistic) OR AUC OR (area under the curve)). This search string incorporated search terms as reported by Hou et al. [12] and Stephan et al. [9] to identify relevant results in a similar way as previous systematic reviews—the reviews by Tang et al. [10, 11] did not report search terms.

We also searched the reference lists from the relevant publications identified in this electronic search. From the systematic reviews, we screened every model that was included. In the electronic search, we filtered based on title and abstract whether a study investigated a prediction model for risk of dementia or AD in non-demented individuals.

A study was eligible when (1) a prediction model was developed in adults ≥ 65 years from the general population without dementia at baseline, (2) the outcome of the prediction model was all-cause dementia or AD, (3) a prediction model included at least age (i.e., the largest risk factor for dementia [15]) as a predictor, and (4) the area under the ROC curve (AUC or concordance (*c*) statistic) in the development cohort was at least 0.70 (values of 0.70–0.80 are acceptable, values of 0.80–0.90 good [16]).

We were unable to externally validate prediction models that were presented without regression coefficients and/or risk scores (i.e., absolute risk) per predictor. Furthermore, we could only calibrate models that included an intercept or baseline hazard. When risk estimates were presented, models without intercept or baseline hazard were recalibrated by estimating their intercept or baseline hazard. We could not validate models for which the predictors or an appropriate proxy was not available in our validation cohort AGES-RS.

**Fig. 1:**
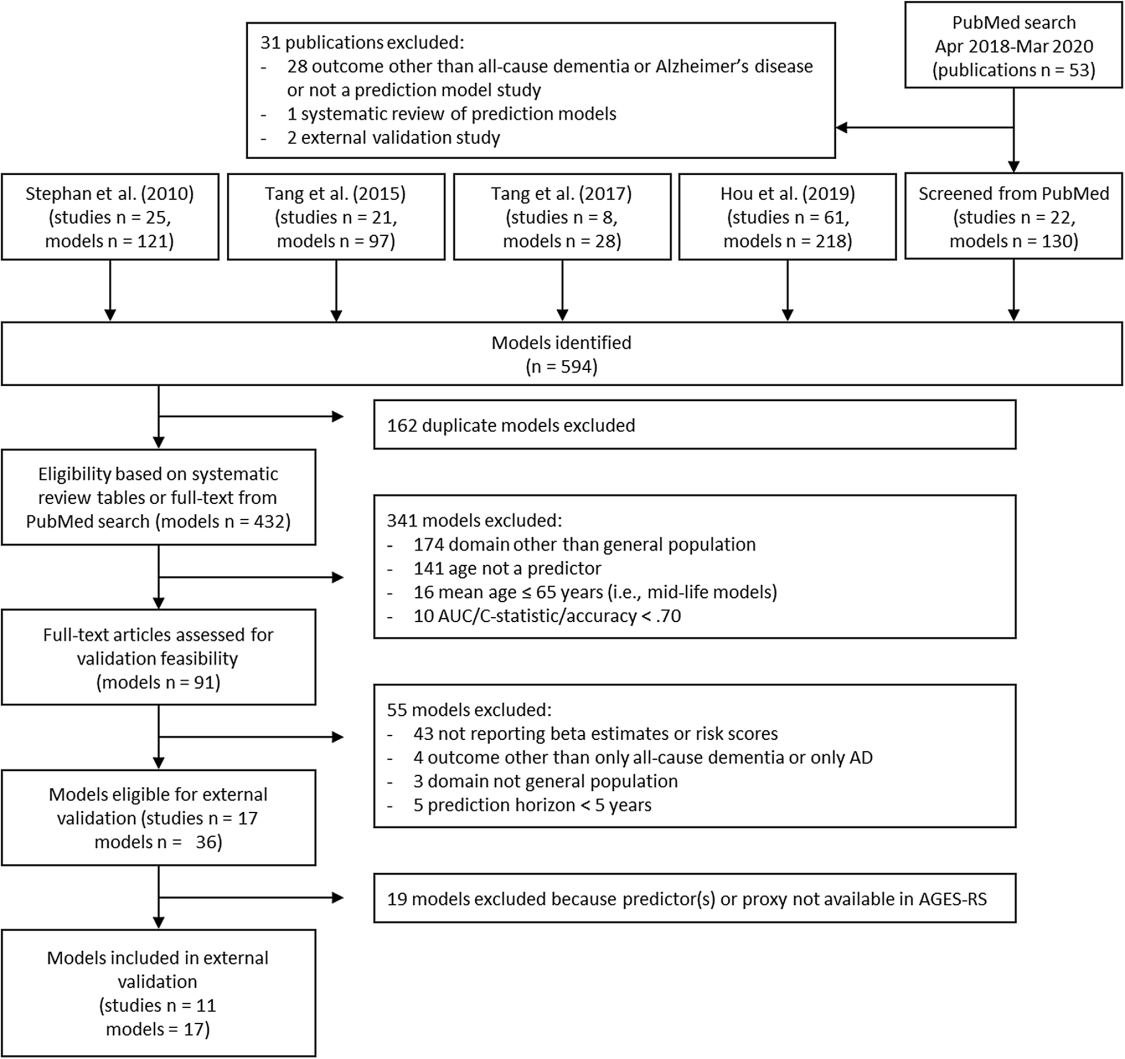
Flowchart of selection of prediction models for risk of all-cause dementia and/or AD in older adults for external validation

In total, we identified 17 all-cause dementia and AD prediction models for external validation (Table 1). A flowchart for model selection is presented in Fig. 1.

**Table 1:** Selected prediction models for validation, description of their development cohorts, and development and validation *c* statistics

Publication	Country of recruitment	Cohort	Sample size	Baseline age	Predicted outcome	Prediction horizon	Model type	Development *c* statistic [95% CI]	Validation *c* statistic [95% CI]
*All-cause dementia*									
5–6 year models									
Anstey et al. [19]* (a)	USA;Sweden	Rush MAP; Kungsholmen Project; CHS-CS	1164; 1301; 3376	62–95, mean 72.3	Dementia	6	Cox	.72 [.68, .76]; .65 [.62, .69]; .73 [.70, .75]	.73 [.71, .76]
Anstey et al. [19]* (b)	USA;Sweden	Rush MAP; Kungsholmen Project; CHS-CS	1164; 1301; 3378	62–95, mean 72.3	Dementia	6	Cox	.68 [.64, .72]; .68 [.64, .71]; .72 [.70, .75]	.71 [.69, .74]
2009 Barnes et al. [25]	USA	CHS-CS	3,375	≥ 65 years	Dementia	6	Logistic	.82 [.79, .84]	.80 [.78, .82]
2014 Barnes et al. [28]	USA	CHS; FHS; HRS; SALSA	2794; 2411; 13,889; 1125	71–73 years	Dementia	6	Cox	.68 [.65, .72]; .77 [.73, .82]; .76 [.74, .77]; .78 [.72, .83]	.72 [.70, .75]
Hogan et al. [24]	Canada	CSHA	892	≥ 65 years	Dementia	5	Logistic	.78 [NA]	.80 [.78, .82]
Li et al. [26]*	USA	FHS	2383	60–88 years	Dementia	5	Cox	.72 [NA]	.71 [.69, .73]
Licher et al. [27]*	Netherlands	Rotterdam Study	2710	≥ 60 years	Dementia	5	Fine & Gray	.79 [.74, .83]	.75 [.73, .77]
10-year models									
Downer et al. [30]	USA	HEPESE	1739	≥ 65 years	Dementia	10	Fine & Gray	.74 [.70, .78]	.72 [.71, .74]
Li et al. [26]*	USA	FHS	2383	60–88 years	Dementia	10	Cox	.72 [NA]	.70 [.68, .72]
Licher et al. [27] *	Netherlands	Rotterdam Study	2710	≥ 60 years	Dementia	10	Fine & Gray	.78 [.75, .81]	.74 [.72, .76]
2010 Tierney et al. [29]	Canada	CSHA	284	≥ 65 years	Dementia	10	Logistic	.79 [NA]	.77 [.76, .79]
*Alzheimer's disease*									
5–6 year models									
Anstey et al. [19]* (a)	USA;Sweden	Rush MAP; Kungsholmen Project; CHS-CS	1164; 1301; 3375	62–95, mean 72.3	AD	6	Cox	.73 [.69, .78]; .64 [.60, .68]; .74 [.71, .77]	.68 [.64, .71]
Anstey et al. [19]* (b)	USA;Sweden	Rush MAP; Kungsholmen Project; CHS-CS	1164; 1301; 3377	62–95, mean 72.3	AD	6	Cox	.69 [.65, .73]; .67 [.63, .70]; .73 [.71, .76]	.67 [.63, .70]
Mura et al. [31]	France	The Three City Study	2558	mean 80 years	AD	5	Logistic	.81 [.76, .87]	.81 [.79, .84]
10-year models									
2005 Tierney et al. [33]	Canada	CSHA	551	≥ 65 years	AD	10	Logistic	.77 [NA]	.76 [.74, .78]
Verhaaren et al. [32] (a)	Netherlands	Rotterdam Study	5507	45–99	AD	10	Cox	.79 [.77, .81]	.70 [.68, .72]
Verhaaren et al. [32] (b)	Netherlands	Rotterdam Study	5507	45–99	AD	10	Cox	.81 [.80, .83]	.73 [.71, .75]

*MAP* memory and aging study, *CHS* cardiovascular health study, *CHS-CS* CHS cognition study, *FHS* framingham heart study, *HRS* health and retirement study, *SALSA* sacramento area latino study on aging; *CSHA* canadian study of health and aging, *HEPESE* hispanic established populations for the epidemiologic study of the elderly, *AD* Alzheimer's disease, Dementia = all-cause dementia; Validation cohort = AGES-RS; Anstey et al. did not use development cohort, based on their own validation

*Model was developed for more than 1 outcome or time horizo

### Independent validation cohort

AGES-RS is a prospective population-based cohort study aimed at investigating risk factors for conditions of old age; the cohort, recruitment, design, and procedures are described in detail elsewhere [17]. In short, AGES-RS is a continuation of the Reykjavik Study, which in 1967 recruited a random sample of 30,795 men and women living in Reykjavik who were born between 1907 and 1935. Between 2002 and 2006, AGES-RS randomly selected 5764 surviving participants from the original Reykjavik Study sample. A follow-up exam (AGES-RS2) was conducted five years later from 2007 to 2011 and included 3316 participants. Event status of participants (diagnosis of dementia or death) was continuously followed-up through nursing home records, medical records, and death certificates until October 2015.

Incident dementia status was assessed at AGES-RS2 following a 3-stage process, which was similar to the ascertainment of prevalent dementia in AGES-RS. The process included (1) a cognitive screening of the total sample; (2) a detailed neuropsychological exam in individuals who screened positive, and a further neurologic and proxy exam in the subset of individuals who also screened positive on test results of the detailed exam; (3) a consensus conference where a neurologist, geriatrician, neuropsychologist and radiographer reviewed relevant data. Additional cases were identified through comprehensive nursing home records, as well as medical records and death certificates following international guidelines for dementia diagnosis. When the individual moved into a nursing home, date of diagnosis was based on the intake exam into the nursing home (all-cause and AD diagnosis); additional incident dementia cases were identified in the nursing home following a standardized protocol followed by all Icelandic nursing homes [18].

The time at risk was measured from entry in the AGES-RS study to diagnosis of dementia, date of death, or end of follow-up (October 2015), whichever came first. In order to account for interval censoring, the time to diagnosis of dementia was calculated as half-way between the last record that reported a diagnosis without dementia and the first record that yielded a diagnosis of all-cause dementia or AD.

Of the 5764 participants at baseline, there were 421 cases of prevalent dementia; as such, our analytic sample of non-demented older adults from AGES-RS was 5343. During 45,021 person-years of follow-up (average follow-up time per person = 8.43 years) 1099 participants developed dementia, and during 40,917 person-years of follow-up 492 participants developed AD.

AGES-RS was approved by the Icelandic National Bioethics Committee (VSN: 00–063), the Icelandic Data Protection Authority (Iceland), and by the Institutional Review Board for the National Institute on Aging, National Institutes of Health (USA). Written informed consent was obtained from all participants.

### Statistical analysis

Participant characteristics in AGES-RS were analyzed using descriptive statistics. Variables in the selected prediction models were matched to variables measured in AGES-RS, or if not available, a proxy variable was used (description of proxies is available in the Supplement). Table 2 lists the predictors in every model. To calculate the risk of dementia for each individual in AGES-RS, we applied the original regression equation for 13 of the 17 models; for 4 models, all from the same report [19], we validated the risk score chart.

**Table 2:** Predictors per model

	All-cause dementia											Alzheimer's disease					
	5–6 years							10 years				5–6 years			10 years		
	Anstey et al. [19]* (a)	Anstey et al. [19]* (b)	2009 Barnes et al. [25]	2014 Barnes et al. [28]	Hogan et al. [24]	Li et al. [26]*	Licher et al. [27]*	Downer et al. [30]	Li et al. [26]*	Licher et al. [27]*	2010 Tierney et al. [29]	Anstey et al. [19]* (a)	Anstey et al. [19]* (b)	Mura et al. [31]	2005 Tierney et al. [33]	Verhaaren et al. [32] (a)	Verhaaren et al. [32] (b)
Number of predictors	12	6	11	7	3	7	4	10	7	4	5	12	6	4	3	2	3
Demographics																	
Age	×	×	×	×	×	×	×	×	×	×	×	×	×	×	×	×	×
Se×/gender	×	×						×			×	×	×	×		×	×
Educational level	×	×		×				×			×	×	×	×	×		
Medical history																	
Stroke				×		×	×		×	×							
Diabetes	×	×		×		×		×	×			×	×				
Pain walking/standing								×									
TIA/mini stroke						×			×								
Cancer						×			×								
Head trauma	×											×					
Coronary bypass surgery			×														
Genetics																	
APOE e4 positive			×														×
Anatomical characteristics																	
White matter disease			×														
Ventricular enlargement			×														
Total intracranial volume (ml)																	
Carotid intima-media thickness			×														
Body mass inde×			×	×		×			×								
Cognition																	
MMSE/3MS			×		×												
Delayed memory recall											×			×	×		
DSST total correct			×								×						
Subjective memory concerns					×		×			×							
Functional																	
Difficulty to dress			×														
Difficulty to walk								×									
Difficulty managing money				×			×			×							
IADL score								×									
Lifestyle																	
Alcohol use	×	×	×									×	×				
Smoking status	×	×										×	×				
Fish intake	×											×					
Physical activity	×											×					
Mental leisure activity	×											×					
Social characteristics																	
Marital status						×			×								
Social leisure activity	×							×				×					
Not having friends								×									
Depression																	
Depressive symptoms	×			×				×				×					

*TIA* transient ischemic attack, *MMSE* mini-mental state e×amination, *3MS* modified mini-mental state, *IADL* instrumental activities of daily living

*Model was developed for more than 1 outcome or time horizon;

Discrimination of each model was calculated with a *c* statistic with 95% confidence interval for 6-year or 10-year risk following the model’s original prediction horizon. Due to the timing of the follow-up assessment, a larger number of cases received an updated diagnosis at si× years rather than at 5 years; therefore, models developed for 5-year risk were validated at 6 years of follow-up for prediction accuracy.

We produced calibration plots to assess the relationship between predicted and e×pected probabilities, with predicted risk divided into five groups. In a calibration plot with grouped observations, more spread between the groups indicates better model performance than less spread [20]. Observed risks in logistic and Co× models were calculated with Kaplan–Meier estimates, and those in Fine & Gray models with cumulative incidence.^1^ The average slope was calculated by using the slope (observed risk divided by predicted risk) across each of the four segments between the cut points. Models that presented a visual mismatch between the predicted and observed risks during calibration were recalibrated by re-estimating the intercept/baseline hazard and calibration slope by multiplying all coefficients with the same estimated factor [21]. No calibration-in-the-large was performed, which only re-estimates the intercept/baseline hazard, as visual inspection indicated the slope also needed to be updated.

For models of which the intercept or baseline hazard was reported, calibration was assessed using the known intercept or baseline hazard to calculate predicted risks. For models of which the intercept or baseline hazard was not reported, the intercept or baseline hazard was first estimated in AGES-RS to calculate predicted probabilities for recalibration. A model’s intercept was estimated by regressing the linear predictor as an offset variable on the event outcome using logistic regression. A model’s baseline hazard was obtained by estimating the baseline survival curve using a Cox model. The models by Anstey et al. [19] were based on risk scores obtained from previous literature, and no predicted risks corresponding to the obtained score were provided; therefore, these models could not be (re)calibrated.

This report is in accordance with the TRIPOD statement (Transparent Reporting of a Multivariable Prediction Model for Individual Prognosis or Diagnosis) [22]. Analyses were performed in R Version 3.5.1; missing data in AGES-RS were handled with multiple imputation analyses in R using the package *mice* [23].

## Results

### Validation population and occurrence of outcome

Descriptive characteristics of the 5343 individuals without dementia at baseline in AGES-RS are presented in Table 3. The mean observed risk of dementia was 10.1% [9.2–11.0] for 6 years and 22.8% [21.5–24.1] for 10 years; the risk for AD was 5.6% [4.9–6.3] and 12.1% [11.0–13.2], respectively.

**Table 3:** Validation sample characteristics of all variables included in at least one model

	Overall	% missing
Demographics	n = 5343	
Age	76.6 (5.7; 66–98)	0.0
Sex/gender (woman)	3097 (58.0)	0.0
Educational level		
Primary	1130 (22.5)	6.2
Secondary	2508 (50.0)	
College	792 (15.8)	
University	582 (11.6)	
Medical history		
Stroke	339 (6.5)	2.0
Diabetes type 2	671 (12.6)	
Intermittent claudication	218 (4.3)	5.2
TIA/mini stroke	213 (4.1)	3.3
Cancer	823 (15.6)	1.3
Head trauma (loss of consciousness)	408 (8.1)	5.7
Coronary bypass surgery	363 (7.1)	3.9
Genetics		
APOE e4 positive	1466 (27.6)	0.4
Anatomical characteristics		
Relative white matter lesion volume	.01 (.01; .00–.13)	18.6
Ventricular CSF volume (ml)	45.22 (20.54; 7.21–182.79)	17.7
Total intracranial volume (ml)	1501.02 (148.18)	18.6
Carotid intima-media thickness	.97 (.14; .60–2.03)	10.0
Body mass index	27.08 (4.45; 13.63–49.70)	0.8
Cognition		
MMSE total score	26.72 (3.01; .00–30.00)	0.6
CVLT delayed recall	6.04 (3.14; .00–16.00)	9.0
DSST total correct	28.91 (10.91; .00–73.00)	3.1
Subjective memory concerns	1586 (30.5)	2.8
Functional		
Difficulty to dress		
No difficulty	4646 (92.7)	6.2
Some difficulty	305 (6.1)	
Much difficulty	51 (1.0)	
Unable	10 (.2)	
Difficulty to walk 500 m		
No difficulty	3854 (74.6)	3.3
Some difficulty	792 (15.3)	
Much difficulty	221 (4.3)	
Unable	301 (5.8)	
Difficulty managing money		
No difficulty	4812 (93.2)	3.3
Some difficulty	168 (3.3)	
Much difficulty	46 (.9)	
Unable	139 (2.7)	
ADL total score	.46 (.95; .00–5.00)	6.3
Lifestyle		
Alcohol use	3345 (65.0)	3.6
Alcohol amount		
1 drink	1523 (45.7)	37.6
2 drinks	1204 (36.1)	
3 drinks	443 (13.3)	
4 or more drinks	166 (5.0)	
Smoking status		
Never	2256 (43.6)	3.2
Former	2281 (44.1)	
Current	635 (12.3)	
Fish intake (%)		
Never	20 (.4)	6.3
Less than once a week	109 (2.2)	
1–2 times a week	1361 (27.2)	
3–4 times a week	3115 (62.2)	
5–6 times a week	352 (7.0)	
Daily	41 (.8)	
More than once a day	9 (.2)	
Physical activity		
Never	2272 (45.6)	6.8
Rarely	804 (16.1)	
Occasionally	343 (6.9)	
Moderate	807 (16.2)	
High	753 (15.1)	
Mental leisure activity (days per month)	6.91 (5.95; .00–30.00)	6.3
Social characteristics		
Marital status		
Married/Living together	3012 (60.1)	6.2
Widow or widower	1439 (28.7)	
Divorced	279 (5.6)	
Single	284 (5.7)	
Social leisure activity (days per month)	3.84 (3.75; .00–18.30)	6.4
Number of close friends	3.39 (3.51; .00–50.00)	6.3
Depression		
Geriatric Depression Scale (15-item)	2.38 (2.10; 0–15)	5.7
AD/All-cause dementia		
Incident dementia	1099 (20.6)	
Incident AD	492 (10.4)	
Follow-up years (incident dementia)	8.43 (3.43; .00–13.37)	
Follow-up years (incident AD)	8.64 (3.43; .00–13.37)	

*AD* Alzheimer’s disease, cells represent mean (SD; range) for continuous variables and number (percentage) for categorical variables; *TIA* transient ischemic attack, *APOE* apolipoprotein E, *CSF* cerebrospinal fluid, *MMSE* mini-mental state examination, *CVLT* california verbal learning test, *DSST* digit symbol substitution test

Models ranged from 2 to 12 predictors (Table 2); predictors could be categorized as demographics, medical history, genetics, anatomical characteristics, cognition, functional, lifestyle, social characteristics, and depression. Table 3 includes the percentage of missing data for each predictor variable in AGES-RS.

### Discrimination and calibration: all-cause dementia

**Fig. 2:**
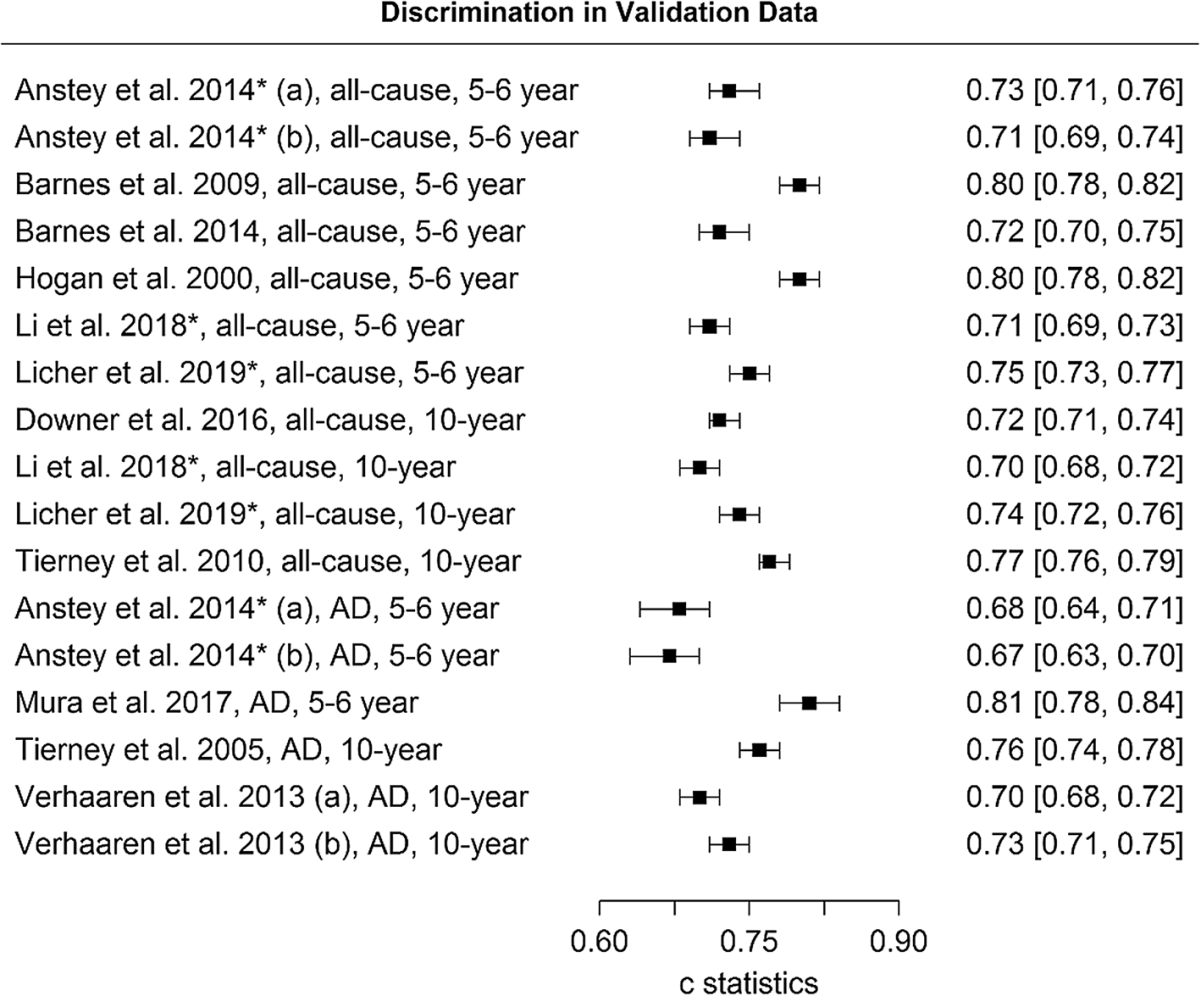
Distribution of *c* statistics across studies with 95% confidence intervals (*model was developed for more than 1 outcome or time horizon)

**Fig. 3:**
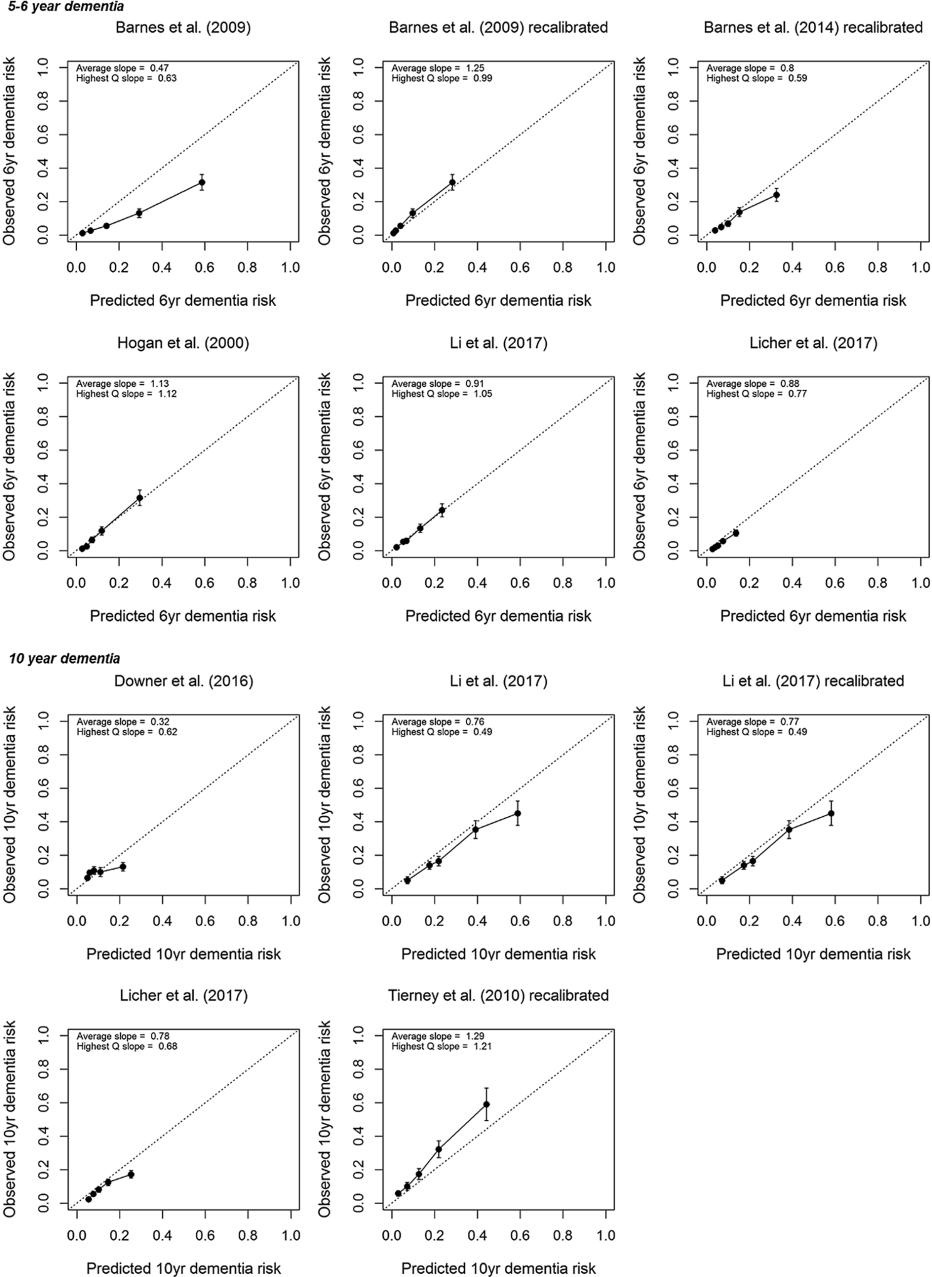

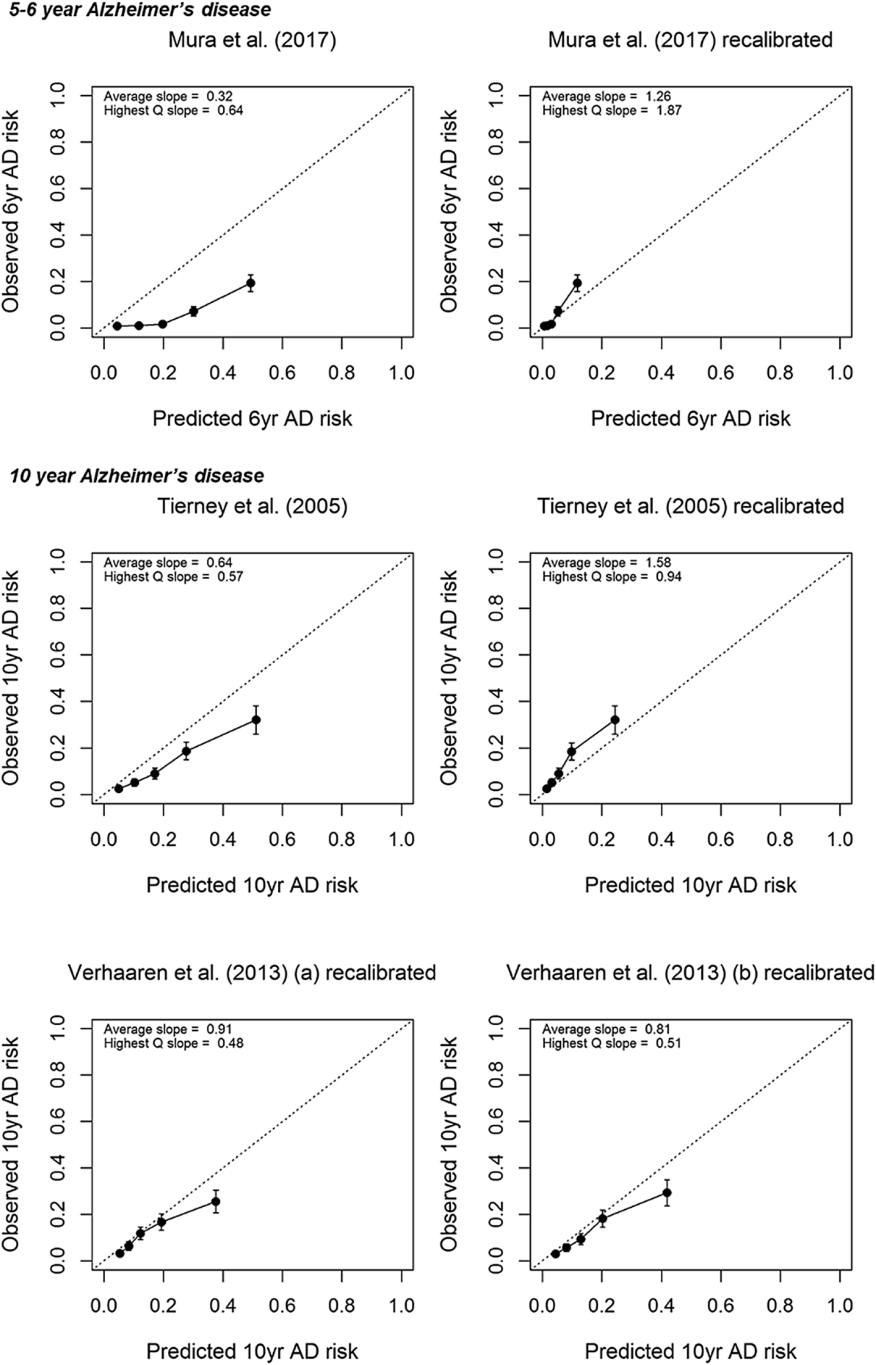
Calibration plots of (re)calibrated prognostic models to predict risk of (**a**) all-cause dementia and (**b**) Alzheimer’s disease; an intercept of 0 and slope of 1 (i.e., the diagonal line) represents ideal calibration and more spread between the groups indicates better model performance than less spread—error bars in grouped observations represent 95% confidence intervals; Q = quartile

Table 1 and Fig. 2 present the validated models for dementia and their *c* statistics with confidence intervals. Figure 3a shows calibration plots including slopes. Among the seven 5/6-year models with all-cause dementia as an outcome, *c* statistics ranged from 0.68 to 0.80; a value of 0.80 was obtained by the models by Hogan et al. [24] and 2009 Barnes et al. [25] Calibration was deemed good for the models by Hogan et al. [24], Li et al. [26], and Licher et al. [27], while the 2009 model by Barnes et al. [25] overestimated the predicted risk. Recalibration of the 2009 model by Barnes et al. [25] improved agreement between observed and predicted risks. Recalibration due to a missing intercept/baseline hazard yielded reasonable calibration for the 2014 model by Barnes et al. [28], although risk was overestimated for the highest risk group.

Among the four 10-year models with all-cause dementia as an outcome, *c* statistics ranged from 0.70 by the model by Li et al. [26] to 0.77 by the model by 2010 Tierney et al. [29]. Calibration for 10-year models was worse compared to the 5/6-year models. Risks were overestimated by the models by Downer et al. [30], Li et al. [26], and Licher et al. [27], particularly in the highest risk group. The 2010 model by Tierney et al. [29] systematically underestimated predicted risks. Recalibration of the model by Li et al. [26] hardly improved the agreement between observed and predicted risks.

### Discrimination & calibration: Alzheimer’s disease

*C* statistics for models with AD outcome including confidence intervals are presented in Table 1, and calibration plots including slopes are shown in Fig. 3b. Among the three 5/6-year models with AD as an outcome, *c* statistics were 0.67 for the model by Anstey et al. [19] that used only common variables, 0.71 for the full model by Anstey et al. [19], and 0.81 for the model by Mura et al. [31]. Only the model by Mura et al. [31] could be calibrated, and showed systematic overestimation of predicted risk compared to observed risk; recalibration improved this relationship but resulted in some degree of underestimation in the highest risk group.

Among the three 10-year models with AD as an outcome, *c* statistics were 0.70 for the simple model by Verhaaren et al. [32] (a) that included age + sex/gender, 0.73 for the model by Verhaaren et al. [32] (b) that included the simple model + APOE e4, and 0.76 for the 2005 model by Tierney et al. [33]. Calibration of the 2005 model by Tierney et al. [33] and recalibration due to missing baseline hazards of the models by Verhaaren et al. [32] showed that all three models overestimated predicted risk, particularly in the highest risk group. Recalibration of the 2005 model by Tierney et al. [33] did not improve the relationship between predicted and observed risks, resulting in systematic underestimation of predicted risk.

### Patterns among predictors

Hierarchically sorting the *c*-statistics from best discriminatory ability to worse revealed that all models with an objective cognitive test obtained *c* statistics ≥ 0.76 (n = 5), all models without ≤ 0.75 (n = 12) (Supplementary Table S1). Additionally, the two models by Licher et al. [27] with subjective memory complaints ranked highest after the models with objective cognition (*c* statistics 0.75 and 0.74)—models without any cognitive measure ranked ≤ 0.73. No clear patterns emerged among other categories of predictors. Additionally, no pattern was observed regarding the number of predictors, prediction outcome, or prediction horizon.

### Complementary validation

For the five best discriminating models (*c* statistics ≥ 0.76) we performed discrimination and calibration for the complementary outcome (all-cause dementia vs. AD) and prediction horizon (6 years vs. 10 years). These models included the ones by Mura et al. [31], 2009 Barnes et al. [25], Hogan et al. [24], 2010 Tierney et al. [29], and 2005 Tierney et al. [33]—calibration was performed with the reported intercept or baseline hazard, which excluded complementary calibration for the 2010 model by Tierney et al. [29].

Discrimination showed that all five models maintained acceptable discriminatory ability (*c* statistics ≥ 0.76) for other combinations of prediction outcome and prediction horizon (Supplementary Table S2). Calibration for other combinations of prediction outcome and prediction horizon showed that, while not developed for that particular prediction outcome and/or horizon, the models by Mura et al. [31], 2009 Barnes et al. [25], and 2005 Tierney et al. [33] all three calibrated best for 10-year dementia risk (Supplementary Figure S1). The model by Hogan et al. [24] calibrated best for the original prediction outcome and horizon of 6-year dementia risk.

## Conclusions

We externally validated 17 out of 36 eligible previously developed prediction models of all-cause dementia or AD for prediction horizons of five to ten years in the population-based AGES-RS cohort. We found acceptable (*c* statistic > 0.70) discriminatory ability to predict who will and who will not develop dementia for nearly all models. Calibration of models ranged from good calibration to poor calibration with systematic overestimation of predicted risks or overestimation for particularly the highest risk group. Recalibration of models often resulted in underestimation of predicted risks, particularly in the highest risk group. We observed a clear pattern that the best discriminating models all included cognition as a predictor; of these, only two models also had good (re)calibration. No clear patterns for discriminatory ability emerged among other categories of predictors, the number of predictors in a model, the prediction outcome, or the prediction horizon. Complementary discrimination of the five best performing models showed that all of them discriminated well for combinations of the other prediction outcome and/or prediction horizon.

When externally evaluating the performance of a prediction model, both discrimination and calibration should be assessed: good performance on one measure does not ensure good performance on the other [34]. Among the models that we externally validated, best performance combining both discrimination and calibration was achieved by the original model by Hogan et al. [24] and the recalibrated 2009 model by Barnes et al. [25] In our validation data, proxies had to be used for several predictors in both models; despite these proxies, the models obtained acceptable discrimination and good (re)calibration.

We observed that the dementia prediction models that included cognition as a predictor showed better discrimination compared to models that did not. Cognitive impairment is one of the core clinical criteria for diagnosis of all-cause dementia and AD [35] and subtle cognitive impairment arises several years before a clinical diagnosis can be established [36, 37]. We should note that general population cohorts, including the AGES-RS cohort, typically subsume individuals across the cognitive spectrum, including those with mild cognitive impairment (MCI). MCI is often considered a symptomatic prodromal phase of dementia that includes mild objective cognitive impairment and subjective complaints while maintaining normal everyday functioning [38, 39]. We should note, however, that MCI has been defined and operationalized in different ways across studies and guidelines [38, 40, 41] and that a subset of individuals with this diagnosis reverts to normal levels in follow-up visits [42, 43]. Nonetheless, the characteristic of cohort studies to include individuals across the cognitive spectrum may (partially) explain the importance of cognition as a predictor for dementia and AD in the general population.

None of the other type of predictor variables (e.g., depression, life style, medical history) showed a similar pattern that distinguished the better performing models from the others—even though the majority of these predictors have consistently been associated with dementia. Several studies have explicitly evaluated the addition of certain types of variables to prediction models of dementia, such as the addition of MRI [44] or genetic data [32], showing that these additions did not improve model performance. In contrast, cognition often does improve performance of a prediction model [31]. Future studies aiming to generate a new prediction model or update an existing one for dementia in the general population should therefore consider cognitive predictors during model development. Among cognitive predictors, it should be investigated which tasks are widely available and cover a range of cognitive functioning to maximize their usefulness with regard to validity and sensitivity. For example, while the Mini-Mental State Examination (MMSE) is a common tool among studies, it suffers from ceiling effects in individuals without dementia. Another consideration when including cognitive predictors would be whether scores should be included as raw scores, normed scores, or sample-adjusted scores for e.g., age, sex/gender, education, race/ethnicity, independent of including these demographic factors into the prediction model.

In general, the evaluated models’ discrimination ability was comparable to the *c* statistic each model obtained in their development cohort—the development *c* statistic was somewhat lower for the majority of models, which is often expected because of optimism in the development data [45]. Nonetheless, three models obtained a better *c* statistic in the AGES-RS data than in (some of) their development data [19, 24, 28]. These deviations demonstrate the different distribution of characteristics in each independent cohort, and highlight the importance of external validation across multiple populations.

External validation for models’ original prediction horizon and original prediction outcome honors that the model parameters were developed for these specific settings. For the purpose of direct comparability, external validation studies sometimes validate models for different outcomes and prediction horizons [8, 46]. Among the validated models in this study, we observed that certain models were developed for multiple horizons and outcomes, and performed approximately equal across different outcomes and horizons [19, 26, 27]. Similarly, we showed in the complementary validation analyses that the best performing models also maintain adequate performance for a different outcome and/or prediction horizon. This transportability of a model across dementia outcomes and prediction horizons adds value to the model for widespread clinical application.

This study adds a uniquely high amount of external validation information, as the number of external validation studies of dementia prediction models is extremely limited [8, 10]. Additional strengths of our study encompass the large sample size of the validation cohort in conjunction with a sizeable number of events, handling of missing data by multiple imputation analyses, and the methodologically complex external validation of competing risk models. The thorough combination of using existing systematic reviews complemented with an updated systematic literature search ensured inclusion of the vast majority of previously published dementia prediction models for consideration in this study.

It is commonly accepted that external validation can be challenging due to limitations in the external dataset regarding available predictors, age range, and prediction horizons [10]: we were unable to validate all 36 models that we initially deemed eligible. Another limitation of this study is the absence of racial and ethnic diversity in the AGES-RS cohort, which is not surprising given that the Icelandic population is almost entirely White from northern European origin [47]. The absence of diversity prohibited evaluation of the models across different race/ethnicity groups, limiting generalizability of the results. Dementia prediction models are typically developed in relatively homogeneous populations that are primarily White and well-educated. Therefore, the field is in high need of more external validation studies of dementia prediction models across a multitude of datasets that are racially/ethnically, culturally, educationally, and geographically diverse.

This study underscored various shortcomings of available dementia prediction models. Multiple models used predictor variables that are typically not widely administered (e.g., time to put on and button a shirt [25]) or entered into a database (e.g., item-level data [48–50]). This specificity of certain variables limits not only external validation but also clinical practice. Notably, while continuous variables typically result in better prediction accuracy, categorizing variables facilitates external validation as in the absence of a direct match, a categorized variable may be substituted by a proxy variable. We also observed that various models for dementia prediction in the general population have been developed for relatively short prediction horizons (e.g., 2–4 years) [31, 51, 52]. Individuals who are only a few years out from a clinical diagnosis of dementia often already have MCI [53]. Therefore, a relatively short prediction horizon to estimate dementia risk in non-demented individuals may not add clinical utility or aid selection for clinical trials, as a diagnosis of MCI already puts an individual at high risk for dementia [53], which renders the prediction model unnecessary. Given the long preclinical phase of dementia and the disappointing results of clinical trials to date, the window of opportunity for preventive interventions and therapeutic trials is necessarily moving to earlier stages than clinical disease manifestation.

Prediction models are clinically valuable to inform individuals and health professionals about dementia risk; however, non-validated prediction models have no assurance they can be applied outside of the study population it was developed on, and should therefore not be used in clinical practice. Models for dementia prediction in the general population that discriminate and calibrate well can aid selection of high-risk individuals for clinical trials and prevention [8]. Our study showed that multiple models showed overestimation of predicted risks during calibration; these models may be acceptable for exclusion of all-cause dementia or AD (if the predicted risk is low, the observed risk is even lower), but lack the ability to accurately identify individuals at higher risk. Future research should aim for additional external validation of existing models, as well as developing new or updating existing models that obtain higher discriminatory ability in both internal validation and independent external validation. While few current dementia prediction models include the competing risks of death and informative censoring, future models will want to incorporate these elements, particularly when using longer prediction horizons. For the development of a potentially widely applicable dementia prediction model, we particularly recommend the inclusion of an objective cognitive test, the use of commonly available variables, inclusion of only a small number of predictors, and the use of a prediction horizon of 10 or more years if the aim is to predict all-cause dementia or AD in the general population.

## Supplementary Information

Below is the link to the electronic supplementary material.

Supplementary file1 (DOCX 632 KB)

## Data Availability

Data are available from the National Institute on Aging (contact via Lenore J. Launer, corresponding author for AGES-RS) for researchers who meet the criteria for access to confidential data.

## Footnotes

1 Note that the model by Downer et al. provided a limited number of predicted risk-categories in a nomogram only for their risk score model—thus, we manually divided individuals into five groups based on the provided predicted risks to obtain five cut points for calibration (n per group: 2027 (≤ 5%), 1280 (> 5–6%), 717 (> 6–8%), 527 (> 8–11%), 792 (> 11%)).
