# Supplementary material for: Dementia risk in the general population: large-scale external validation of prediction models in the AGES-Reykjavik study
Source: Eur J Epidemiol. 2021 Jul 25;36(10):1025–41. doi: 10.1007/s10654-021-00785-x (PMC8542560; doi:10.1007/s10654-021-00785-x)
Supplement: Supplementary file 1 — Supplementary file1 (DOCX 632 KB) [file 10654_2021_785_MOESM1_ESM.docx]

# Supplementary material

## Supplementary Text 1. Proxy variables

A proxy variable was used if no direct match for a predictor was available in the AGES-RS data; a direct match was available for all predictors in the models by Li et al. [1] and Verhaaren et al. [2].

In AGES-RS, education is measured in four levels following the educational system in Iceland. For models that used education as a continuous variable [3-5], we recoded education level as follows: primary school = 6 years of education, secondary school = 12 years of education, college = 16 years of education, and university = 18 years of education. For the model by Mura et al. [6], which uses levels of education based on the French educational system, we recoded education as follows: primary school = French junior-school diploma, secondary school = vocational school certificate and French high-school diploma, college = graduate studies, and university = graduate studies. For the model by Downer et al. [7], low education (having completed < 4 years of education) was coded as having completed only primary school.

For models that used the Rey Auditory Verbal Learning Test (RAVLT [8]) to measure delayed memory recall [3, 4], a 15-word list learning task, we rescaled performance on the California Verbal Learning Test (CVLT [9]), a 16-word list learning task used in AGES-RS, to a minimum of 0 and a maximum of 15. For models that used the Modified Mini-Mental State (3MS [10]) [11, 12], we rescaled performance on the MMSE by dividing the MMSE score by 30 and multiplying the result by 100. For models that used depressive symptoms as a predictor [5, 7, 13], we used the 15-item Geriatric Depression Scale (GDS) as a proxy variable, coded negative for less than 6 points versus positive for 6 or more points. For models that used as a predictor requiring assistance with money or medications [13, 14], we used a proxy in AGES-RS regarding how difficult it is to manage money (expense/bill), for which we dichotomize the answers between ‘no difficulty’ or ‘some difficulty’ versus ‘much difficulty’ and ‘I am unable to do it.’

The 2009 model by Barnes et al. [12] included a dichotomized variable that indicated the time one needs to put on and button a shirt (≤ 45 seconds or > 45 seconds). We used a proxy variable in AGES-RS regarding how difficult it is to dress (tie/zippers/buttons) as a proxy, for which we dichotomize the answers between ‘no difficulty’ or ‘some difficulty’ versus ‘much difficulty’ and ‘I am unable to do it.’ As a proxy for white matter disease on MRI when higher than grade 3 on an ad hoc scale from 0 to 9 [15], we used relative white matter brain volume at a median split. As a proxy for enlarged ventricles on MRI when higher than grade 4 on an ad hoc scale from 0 to 9 [15], we used ventricular CSF volume relative to ICV, which we dichotomized at a median split. As a proxy for carotid artery maximum thickness, we used carotid artery mean thickness, which we split at the third quartile.

The models by Anstey et al. [5] included the predictor cognitively stimulating activities, for which we used a variable in AGES-RS that represented mental leisure activities as measured in days per month; low cognitively stimulating activities was coded as three or fewer days per month, cognitively stimulating activities was coded as more than three but fewer than 15 days per month, and high cognitively stimulating activities was coded as 15 or more days per month. As a proxy for social network, we used a variable that represented social leisure activities as measured in days per month; low social network was coded as zero days per month, medium-low social network was coded as one to six days a month, medium-high social network was coded as more than six but fewer than nine days per month, and high social network was coded as nine or more days per month. As a proxy for levels of alcohol consumption (abstainers, light-to-moderate, heavy), we recoded number of drinks per day to 0 for abstainers, one to two per day for light-to-moderate, and more than two per day as heavy. As a proxy for physical activity, we used a variable in AGES-RS regarding the frequency of moderate to vigorous physical activity in the past 12 months; we recoded never, rarely, and occasionally as low physical activity, moderate as medium physical activity, and high as high physical activity. As a proxy for fish intake in servings a week, we recoded how often one eats fish as a main meal as follows: never or less than once a week = 0-.25 servings a week, 1-2 times a week = .26-2 servings a week, 3-4 times a week = 2.1-4 servings a week, and 5-6 times a week, daily, and more than once a day = 4.1 or more servings a week.

The model by Downer et al. [7] included not having friends to count on as a predictor, for which we used the AGES-RS variable of number of friends you feel close to and dichotomized it as 0 versus 1 or more. As a proxy for not attending community events, we used social leisure activities as measured in days per month and dichotomized it as 0 versus 1 or more days. As a proxy for instrumental activities of daily living (IADL) we used activities of daily living (ADL). Lastly, as a proxy for ability to walk a half-mile, we used ability to walk 500 meters.

Supplementary Figure S1. Calibration plots for other prediction outcome and/or prediction horizon (outlined in red are original calibration plots)


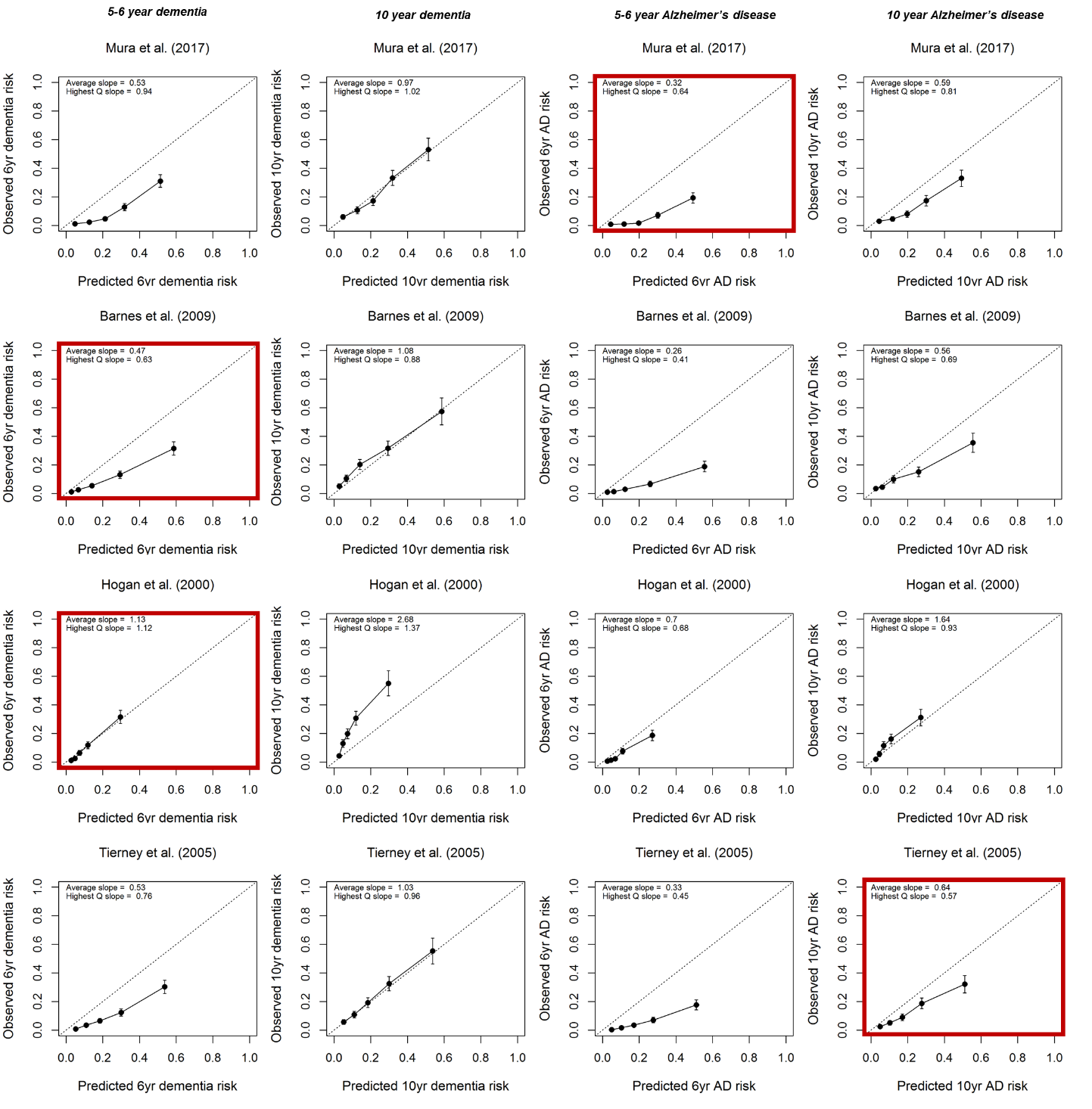


Supplementary Table S1. Pattern by predictor type when ranking models by *c* statistic from high to low

| **Model** | **Prediction outcome** | **Prediction horizon** | **C-statistic validation** | **Predictors**  **number** | **Demo-graphics** | **Medical history** | **Genetics** | **Anatomical characteristics** | **Cognition** | **Functional** | **Lifestyle** | **Social characteristics** | **Depression** |
| --- | --- | --- | --- | --- | --- | --- | --- | --- | --- | --- | --- | --- | --- |
| Mura et al. [6] | AD | 5-6 years | .81 | 4 | Yes | No | No | No | Yes (O) | No | No | No | No |
| 2009 Barnes et al. [12] | Dementia | 5-6 years | .80 | 11 | No | Yes | Yes | Yes | Yes (O) | Yes | Yes | No | No |
| Hogan et al. [11] | Dementia | 5-6 years | .80 | 3 | No | No | No | No | Yes (O+S) | No | No | No | No |
| 2010 Tierney et al. [4] | Dementia | 10 years | .77 | 5 | Yes | No | No | No | Yes (O) | No | No | No | No |
| 2005 Tierney et al. [3] | AD | 10 years | .76 | 3 | Yes | No | No | No | Yes (O) | No | No | No | No |
| Licher et al. [14]* | Dementia | 5-6 years | .75 | 4 | No | Yes | No | No | Yes (S) | Yes | No | No | No |
| Licher et al. [14]* | Dementia | 10 years | .74 | 4 | No | Yes | No | No | Yes (S) | Yes | No | No | No |
| Verhaaren et al. [2] (b) | AD | 10 years | .73 | 3 | Yes | No | Yes | No | No | No | No | No | No |
| Anstey et al. [5]* (a) | Dementia | 5-6 years | .73 | 12 | Yes | Yes | No | No | No | No | Yes | Yes | Yes |
| Downer et al. [7] | Dementia | 10 years | .72 | 10 | Yes | Yes | No | No | No | Yes | No | Yes | Yes |
| 2014 Barnes et al. [13] | Dementia | 5-6 years | .72 | 7 | Yes | Yes | No | Yes | No | Yes | No | No | Yes |
| Li et al. [1]* | Dementia | 5-6 years | .71 | 7 | No | Yes | No | Yes | No | No | No | Yes | No |
| Anstey et al. [5]* (a) | AD | 5-6 years | .71 | 12 | Yes | Yes | No | No | No | No | Yes | Yes | Yes |
| Verhaaren et al. [2] (a) | AD | 10 years | .70 | 2 | Yes | No | No | No | No | No | No | No | No |
| Li et al. [1]* | Dementia | 10 years | .70 | 7 | No | Yes | No | Yes | No | No | No | Yes | No |
| Anstey et al. [5]* (b) | Dementia | 5-6 years | .68 | 6 | Yes | Yes | No | No | No | No | Yes | No | No |
| Anstey et al. [5]* (b) | AD | 5-6 years | .67 | 6 | Yes | Yes | No | No | No | No | Yes | No | No |

Note. *Model was developed for more than 1 outcome or time horizon; AD = Alzheimer’s disease; dementia = all-cause dementia; O = objective; S = subjective

Supplementary Table S2. Complementary discrimination for other prediction outcome and/or prediction horizon

| *Model* | *6-year dementia* | *10-year dementia* | *6-year AD* | *10-year AD* |
| --- | --- | --- | --- | --- |
| Mura et al. [6] | .80 [.78, .82] | .76 [.74, .78] | **.81 [.78, .84]** | .77 [.75, .80] |
| 2009 Barnes et al. [12] | **.80 [.78, .82]** | .77 [.75, .78] | .79 [.76, .82] | .76 [.74, .78] |
| Hogan et al. [11] | **.80 [.78, .82]** | .76 [.75, .78] | .80 [.77, .83] | .76 [.74, .79] |
| 2010 Tierney et al. [4] | .81 [.79, .83] | **.77 [.76, .79]** | .81 [.79, .84] | .78 [.76, .80] |
| 2005 Tierney et al. [3] | .78 [.76, .80] | .76 [.74, .77] | .78 [.75, .80] | **.76 [.74, .78]** |

*Note*. In bold is the original model's prediction horizon and outcome; dementia = all-cause

dementia; AD = Alzheimer's disease

## References Supplementary Materials

1. Li J, Ogrodnik M, Devine S, Auerbach S, Wolf PA, Au R. Practical risk score for 5-, 10-, and 20-year prediction of dementia in elderly persons: Framingham Heart Study. Alzheimer's & Dementia. 2018;14(1):35-42.

2. Verhaaren BF, Vernooij MW, Koudstaal PJ, Uitterlinden AG, van Duijn CM, Hofman A, et al. Alzheimer's disease genes and cognition in the nondemented general population. Biological psychiatry. 2013;73(5):429-34.

3. Tierney MC, Yao C, Kiss A, McDowell I. Neuropsychological tests accurately predict incident Alzheimer disease after 5 and 10 years. Neurology. 2005;64(11):1853-9.

4. Tierney MC, Moineddin R, McDowell I. Prediction of all-cause dementia using neuropsychological tests within 10 and 5 years of diagnosis in a community-based sample. Journal of Alzheimer's Disease. 2010;22(4):1231-40.

5. Anstey KJ, Cherbuin N, Herath PM, Qiu C, Kuller LH, Lopez OL, et al. A self-report risk index to predict occurrence of dementia in three independent cohorts of older adults: the ANU-ADRI. PLoS One. 2014;9(1).

6. Mura T, Baramova M, Gabelle A, Artero S, Dartigues J-F, Amieva H, et al. Predicting dementia using socio-demographic characteristics and the Free and Cued Selective Reminding Test in the general population. Alzheimer's research & therapy. 2017;9(1):21.

7. Downer B, Kumar A, Veeranki SP, Mehta HB, Raji M, Markides KS. Mexican‐American Dementia Nomogram: Development of a Dementia Risk Index for Mexican‐American Older Adults. Journal of the American Geriatrics Society. 2016;64(12):e265-e9.

8. Rey A. L'examen psychologique dans les cas d'encephalopathie traumatique. Arch Psychologie. 1941;28:286-340. PubMed PMID: 518.

9. Delis DC, Kramer JH, Kaplan E, Ober BA. California Verbal Learning Test: Research edition. San Antonio: Psychological Corporation; 1987.

10. Teng EL, Chui HC. The Modified Mini-Mental State (3MS) examination. Journal of Clinical Psychiatry. 1987;48:314-8. PubMed PMID: 1638.

11. Hogan DB, Ebly EM. Predicting who will develop dementia in a cohort of Canadian seniors. Canadian journal of neurological sciences. 2000;27(1):18-24.

12. Barnes D, Covinsky K, Whitmer R, Kuller L, Lopez O, Yaffe K. Predicting risk of dementia in older adults: The late-life dementia risk index. Neurology. 2009;73(3):173-9.

13. Barnes DE, Beiser AS, Lee A, Langa KM, Koyama A, Preis SR, et al. Development and validation of a brief dementia screening indicator for primary care. Alzheimer's & Dementia. 2014;10(6):656-65. e1.

14. Licher S, Leening MJ, Yilmaz P, Wolters FJ, Heeringa J, Bindels PJ, et al. Development and validation of a dementia risk prediction model in the general population: an analysis of three longitudinal studies. American Journal of Psychiatry. 2019;176(7):543-51.

15. Bryan RN, Manolio T, Schertz L, Jungreis C, Poirier V, Elster A, et al. A method for using MR to evaluate the effects of cardiovascular disease on the brain: the cardiovascular health study. American journal of Neuroradiology. 1994;15(9):1625-33.
